# Further studies on *Boreonectes* Angus, 2010, with a molecular phylogeny of the Palaearctic species of the genus

**DOI:** 10.3897/CompCytogen.v11i2.11980

**Published:** 2017-03-22

**Authors:** Robert B. Angus, Ignacio Ribera, Fenglong Jia

**Affiliations:** 1 Department of Life Sciences (Insects), The Natural History Museum, Cromwell Road, London SW7 5BD, UK; 2 Institut de Biologia Evolutiva (CSIC-UPF), Passeig Maritim de la Barceloneta, 37-49, 08003 Barcelona, Spain; 3 Institute of Entomology, Life Science School, Sun Yat-sen University, Guangzhou, 570275, Guangdong, China

**Keywords:** Dytiscidae, *Boreonectes*, *B. emmerichi*, *B. alpestris*, molecular phylogeny, diversity, karyotypes

## Abstract

Karyotypes are given for *Boreonectes emmerichi* (Falkenström, 1936) from its type locality at Kangding, China, and for *B. alpestris* (Dutton & Angus, 2007) from the St Gotthard and San Bernardino passes in the Swiss Alps. A phylogeny based on sequence data from a combination of mitochondrial and nuclear genes recovered western Palaearctic species of *Boreonectes* as monophyletic with strong support. *Boreonectes emmerichi* was placed as sister to the north American forms of *B. griseostriatus* (De Geer, 1774), although with low support. The diversity of Palaearctic species of the *B. griseostriatus* species group is discussed.

## Introduction

The genus *Boreonectes* Angus, 2010 comprises small diving beetles and most of the species are endemic to the Nearctic Region (Angus 2010b). However, one species-complex, including the type species of the genus (*B. griseostriatus* (De Geer, 1774)) is widely distributed in the Palaearctic, where a number of chromosomally distinct species have been recognised (Dutton and Angus 2007; Angus 2008, 2010a; Angus et al. 2015). Angus et al. (2015), in their analysis of *Boreonectes emmerichi* (Falkenström, 1936), noted that the type material, from the Kangding area of Sichuan, China, was darker than the Qinghai material they used for chromosome analysis, and in particular none of the Qinghai material had the darks marks on the pronotum as extensive as in the type material. Nevertheless, they noted that DNA (COI) data obtained by Ignacio Ribera from this material closely matched those obtained from material collected near Nam Tso (Xizang), much further south on the Plateau than the Qinghai material, and in the light of this felt that the Qinghai material could safely be referred to *B. emmerichi*. They suggested that the darker colouration of the Kangding material was perhaps associated with a more wooded environment.

In June–July 2016 one of us (R. B. Angus) had the opportunity to visit the Kangding area and collect *B. emmerichi* from its type area, for chromosome analysis. This confirms the identity of the Qinghai material as *B. emmerichi*. Then in August 2016 R. B. Angus was able to visit Switzerland and to collect *Boreonectes* from the St Gotthard and San Bernardino passes, localities where I. Ribera & A. Cieslak had in 2002 collected material considered to be *B. griseostriatus* (De Geer, 1774) (Abellán et al. 2013) Surprisingly, according to their karyotypes the Swiss populations were found to be *B. alpestris* (Dutton & Angus, 2007), although the sequenced markers were found to have identical sequences to those obtained from the 2002 material.

This study aims to clarify the identities of *B. emmerichi* (Falkenström, 1936) and *B. alpestris* Dutton & Angus, 2007, from some localities in the Alps using karyotype and molecular data. To establish their phylogenetic positions, we build a molecular phylogeny of the genus *Boreonectes* including most Palaearctic and a representation of Nearctic species.

## Material and methods

The material used for chromosome preparations is listed in Table 1.

Specimens were brought back to the laboratory alive and placed in small aquaria where they were fed with living larvae of Chironomidae (Diptera). Chromosome preparation and photography were as described by Dutton and Angus (2007). In fact, most of the *B. emmerichi* died before they reached the laboratory but sufficient material, all from the same locality, survived to give chromosome preparations. Survival of the Swiss *B. alpestris* was much better, but only two specimens gave preparations from which karyotypes could be assembled.

**Table 1.** Material giving chromosome preparations.

Species	Locality	Material
*B. emmerichi* (Falkenström, 1936)	CHINA Sichuan. Kangding County Yalashenshan.30.215°N,101.757°E Small pools 4052 m a.s.l.	2 ♂♂, 4 ♀♀
*B. alpestris* (Dutton & Angus, 2007)	SWITZERLAND Ticino. San Bernardino pass. 46.499°N 101.755°E. Pool 2030 m a.s.l.	1♀
	SWITZERLAND Ticino. St Gotthard pass 46.559°N 8.562°E Pool 2112 m a.s.l.	1♂

For DNA extraction and sequencing we used the same methodology as various recent works on the same family Dytiscidae (e.g. García-Vázquez et al. 2016). Briefly, specimens were directly preserved in absolute ethanol in the field, and preserved at -20°C until processed. Extractions of single specimens were non–destructive, using the DNeasy Tissue Kit (Qiagen GmbH, Hilden, Germany). Vouchers and DNA samples are kept in the collections of the Institut de Biologia Evolutiva (IBE). We included examples of all known species of Palaearctic *Boreonectes* with the sole exception of *B. inexpectatus* (Dutton & Angus, 2007), known from a single lake in the Alps (Dutton and Angus 2007). We also included as outgroups several species of Nearctic *Boreonectes*, including specimens of *B. griseostriatus* (De Geer, 1774) (Table 2). The tree was rooted in two species of the genus *Stictotarsus*, the closest relatives of *Boreonectes* according to the phylogeny in García-Vázquez et al. (2016).

We sequenced four mitochondrial genes in two PCR reactions: 3’ end of cytochrome c oxidase subunit (COI); and a single fragment including the 3’ end of the large ribosomal unit (16S), the whole tRNA–Leu gene and the 5’ end of the NADH dehydrogenase 1. We also amplified fragments of one nuclear gene, histone 3 (H3) (see García-Vázquez et al. 2016 for the primers used). New sequences have been deposited in the EMBL database with Accession Numbers LT796523–LT796555 (Table 2).

**Table 2.** Material studied for the molecular phylogeny, with voucher number, locality and collector, and EMBL accession numbers. In bold, sequences newly obtained for this study.

No	Species	Voucher	Country	Locality	Leg	COI	16S+tRNA +NAD1	H3
1	*B. alpestris* (Dutton & Angus, 2007)	IBE-RA263	Italy	Piemonte. Gran Paradiso Nat. Park. Colle del Nivolet, roadside lake at ca 2500 m. 3.9.2010	R.B. Angus	**LT796525**	**LT796551**	**LT796537**
2	*B. alpestris*	IBE-AN579	Switzerland	Col de San Bernardino, ponds	R.B. Angus	**LT796523**	-	**LT796535**
3	*B. alpestris*	IBE-AN580	Switzerland	St. Gottardo pass, summit	R.B. Angus	**LT796524**	-	**LT796536**
4	*B. alpestris*	MNCN-AI298	Switzerland	Col de San Bernardino, ponds 13.10.2002	I. Ribera & A. Cieslak	HF931186	HF931409	-
5	*B. alpestris*	NHM-IR309	Switzerland	St. Gottardo pass, summit, 27.7.00	I. Ribera & A. Cieslak	HF931275	HF931512	-
6	*B. emmerichi* (Falkenström, 1936)	IBE-AN581	China	Sichuan, Kangding County, Yalashenshan. Small pools 4052m 27.6.2016	R.B. Angus, F-L Jia, Z-Q. Li & K. Chen	**LT796531**	-	**LT796541**
7	*B. emmerichi*	IBE-AN582	China	CHINA Sichuan.Kangding County.Yalashenshan, Small pools 4074 m a.s.l. 27.6.2016	R.B. Angus, F-L Jia, Z-Q. Li & K. Chen	**LT796528**	-	**LT796538**
8	*B. emmerichi*	IBE-AN583	China	Sichuan, Kangding County Boij ta Car parking zone, Grassy and weedy pools. 3531 m a.s.l. 28.6.2016	R.B. Angus, F-L Jia, Z-Q. Li & K. Chen	**LT796529**	-	**LT796539**
9	*B. emmerichi*	IBE-AN584	China	Sichuan, Kangding County Yajiaheng, Shallow stony & peaty pools 3337 m a.s.l. 30.6.2016	R.B. Angus, F-L Jia, Z-Q. Li & K. Chen	**LT796530**	-	**LT796540**
10	*B. emmerichi*	IBE-RA1167	China	N. Qinghai Hu, Gangca, 1 km SE of Gangca Dasi, stream, 3464m 5.6.2013	R.B. Angus, F.L. Jia & Y. Zhang.	**LT796526**	**LT796555**	-
11	*B. emmerichi*	IBE-RA1168	China	Qinghai, Golo, Maduo, Roadside pools on river flats ca 20 km SE Maduo. 8.6.2013	R.B. Angus, F.L. Jia & Y. Zhang.	**LT796527**	-	-
12	*B. emmerichi*	IBE-RA891	Tibet	S Tibet, S Namtso lake 4750m, banks, 21.7.2010	J. Schmidt	**LT796532**	**LT796552**	**LT796542**
13	*B. funereus* (Crotch, 1873)	MNCN-AI1208	California (US)	California, 9.2000	Y. Alarie	HF931173	HF931393	**LT796543**
14	*B. griseostriatus* (De Geer, 1774)	MNCN-AI952	Sweden	prov. Angermanland, Hörnefors parish, Norrbyskäv island, rock pool, 23.6.2006	A.N. Nilsson	**LT796533**	**LT796553**	**LT796544**
15	*B. griseostriatus* (De Geer, 1774) cplx	MNCN-AI1160	California (US)	Napa Co., Knoxville Recreation Area, 2000	T. Berendonk	HF931168	HF931387	**LT796547**
16	*B. griseostriatus* cplx	MNCN-AI1150	California (US)	Sacramento Co., Clay Station Rd., 20.6.1999	W.D. Shepard & C.B. Barr	HF931166	HF931385	**LT796545**
17	*B. griseostriatus* cplx	IBE-RA483	California (US)	Marin Co., Seasonal Pools in Dillans Beach Dunes, Spring 2011.	D. Post	HF931317	HF931541	**LT796546**
18	*B. griseostriatus strandi* (Brinck, 1943)	MNCN-AI1082	Norway	Bugöynes, 29.7.2006	S. Ligaard & B. Andrén	HF931153	HF931372	**LT796548**
19	*B. ibericus* (Dutton & Angus, 2007)	NHM-IR22	Portugal	Sa. Da Estrela, Torre, lagoon 25.7.1998	I. Ribera	EF670064	EF670030	EF670157
20	*B. macedonicus* (Georgiev, 1959)	MNCN-AI1120	Macedonia	Macedonia, Sar (Shar) Planina, Karanikolicko ezero Black Nick’s Lake, 6.9.2006	R.B. Angus	**LT796534**	**LT796554**	**LT796549**
21	*B. multilineatus* (Falkenström, 1922)	MNCN-AI115	Faroes (Isl.)	20.9.2004	J. Hansen	HF931165	HF931384	-
22	*B. riberae* (Dutton & Angus, 2007)	MNCN-AI829	Turkey	Düzce, between Kartalkaya and Çaydurt, pools in mountain pass, 1700m 23.4.2006	I. Ribera	HF931232	HF931461	**LT796550**
23	*B. striatellus* (Le Conte, 1852)	NHM-IR295	California (US)	Mono co., Long Valley 19.6.2000	I. Ribera & A. Cieslak	HF931274	HF931511	-
24	*Stictotarsus falli* Nilsson, 2001	NHM-IR334	New Mexico (US)	New Mexico, 9.2000	Y. Alarie	EF670063	EF670029	EF670155
25	*Stictotarsus roffii* (Clark, 1862)	NHM-IR335	Texas (US)	Texas, 9.2000	Y. Alarie	AJ850607	AJ850355	EF670158

We aligned the sequences using the MAFFT online v.6 and the Q–INS–i algorithm (Katoh and Toh 2008). We used Maximum Likelihood as implemented in RAxML-HPC2 (Stamatakis et al. 2008) in the CIPRES science gateway (Miller et al. 2010), using GTR+G as evolutionary model and three partitions corresponding to the three amplified fragments. Node support was assessed with 100 fast bootstrap replicas.

## Results and discussion

### *B. alpestris* (Dutton & Angus, 2007)

**Figure 1.**
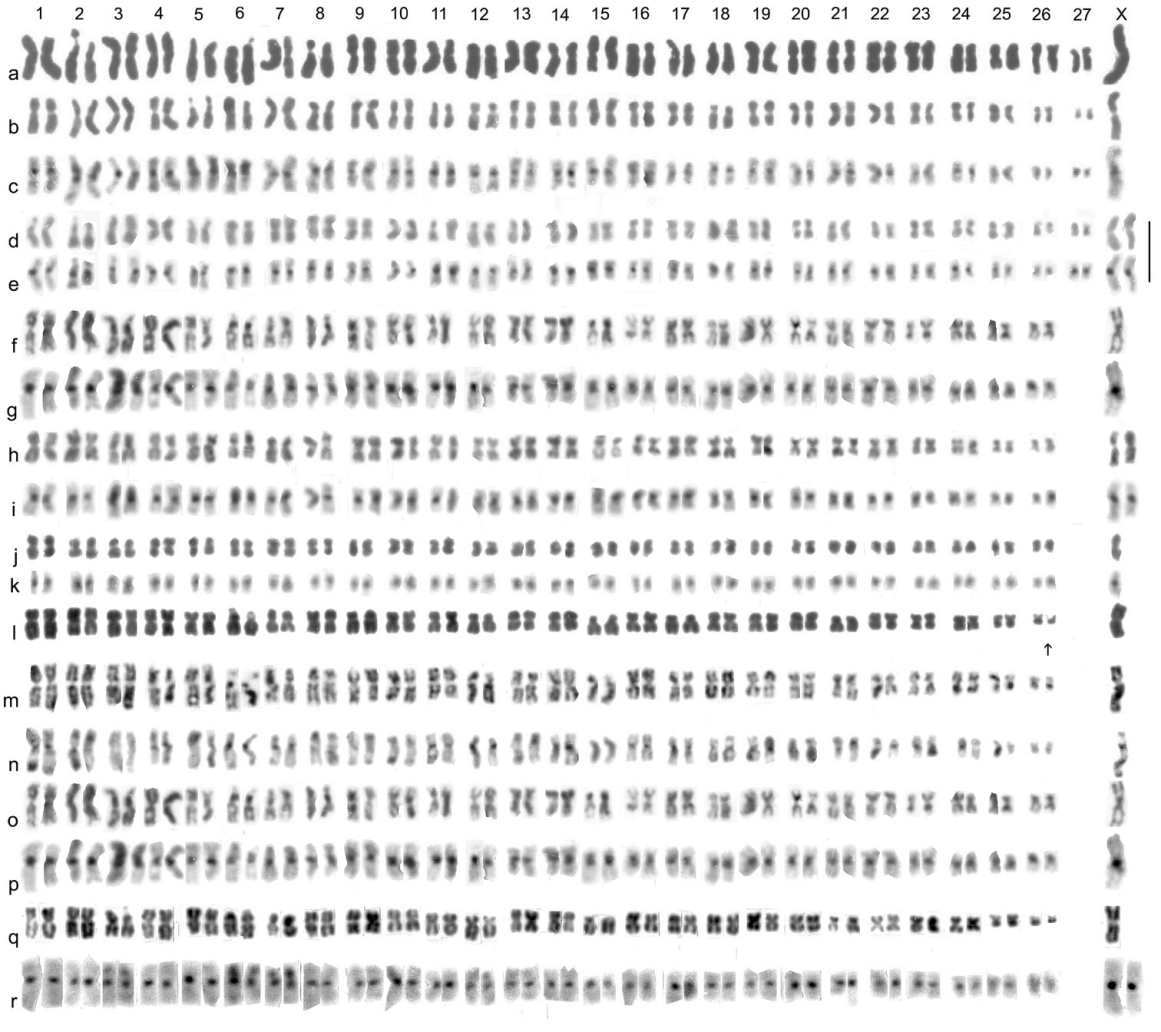
Mitotic chromosomes from mid gut of *Boreonectes* spp., arranged as karyotypes. **a**–**e** *B. alpestris*, **a** paratype ♂, Giemsa stained (from Dutton, Angus, (2007)) **b**, **c** ♂, Colle del Nivolet **b** Giemsa stained, **c** the same nucleus C-banded (from Angus (2010)) **d**, **e** San Bernardino, ♀, **d** Giemsa stained **e** the same nucleus C-banded **f**–**k** *B. emmerichi* **f**, **g** ♂ Yalashenshan **f** Giemsa stained **g** the same nucleus C-banded **h, i** ♀ Yalashenshan **h** Giemsa stained **g** the same nucleus C-banded **j**, **k** ♂ Mado **j** Giemsa stained **k** the same nucleus C-banded (from Angus et al (2015)) **l**–**n** *B. macedonicus* ♂♂ (from Angus (2008)) **l** Crno ezero Giemsa stained **m** Karanikoličko ezero Giemsa stained **n** the same nucleus C-banded **o, p** *B. emmerichi* ♂ shown in **f, g q, r** *B. macedonicus* Crete **q** ♂Giemsa stained **r** ♀C-banded (from Angus (2008). Scale = 5 μm.

**Figure 2.**
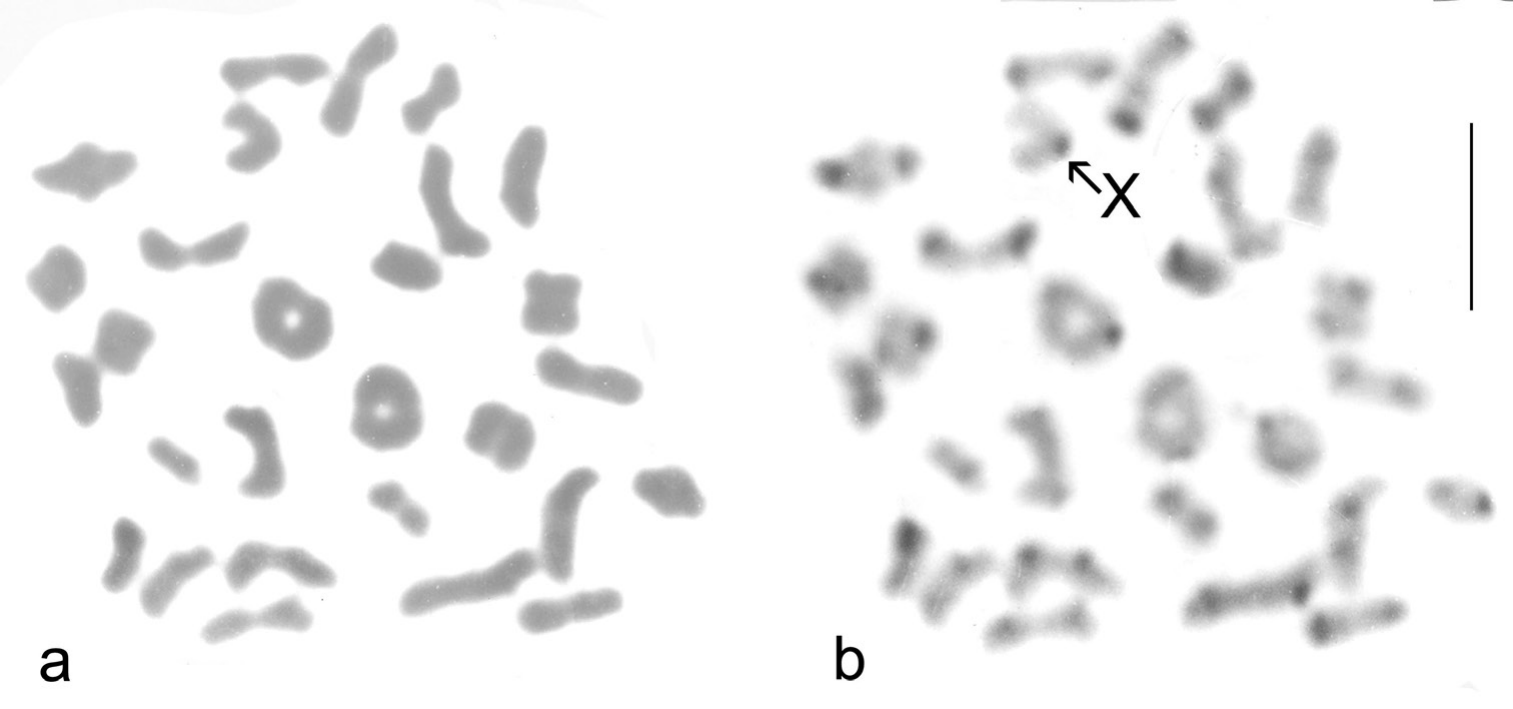
*B. alpestris* ♂ St Gotthard meiosis metaphase I **a** Giemsa stained **b** the same nucleus C-banded. Scale = 5 μm.

Published information: 2n = 54 + X0 (♂), XX (♀) (Dutton and Angus 2007). Mitotic chromosomes, arranged as karyotypes, are shown in Fig. 1a–e. Fig. 1a shows a paratype male, Giemsa stained, Fig. 1b, c shows a male from the Colle del Nivolet, the same nucleus Giemsa stained and C-banded, and Fig. 1d, e shows the same nucleus from a female from the Paso San Bernardino, Giemsa stained and C-banded. The chromosomes in these preparations are in complete agreement with each another. Metaphase I of meiosis from a male from the St Gotthard Pass is shown in Fig. 2a (Giemsa stained) and b (the same nucleus C-banded). The C-banded preparation enables the unpaired X chromosome to be identified. Although preparations from only two of the specimens attempted resulted in complete karyotypes, several other specimens yielded almost complete karyotypes and in no case were more than 55 (♂) or 56 (♀) chromosomes counted. Since *B. griseostriatus* has a karyotype with 61 or 62 chromosomes (♂, ♀) it is very unlikely that this species was present in either sample.

We extracted and sequenced two specimens of *B. alpestris* from the same St. Gotthard and San Bernardino populations used to obtain the karyotypes, which had almost identical sequences for the mitochondrial genes (with only 1 mismatch in the COI gene) as two specimens from the same localities collected in 2000 and 2002 respectively (Table 2) and reported as *S. griseostriatus* in Abellán et al. (2013). These four specimens differ from one sequenced *B. alpestris* from Colle del Nivolet in five nucleotides in the COI gene (with a length of 826), and one in the 16S gene (with a length of 796 nucleotides). The nuclear gene H3 (with a length of 328 nucleotides) was identical for all sequenced specimens of *B. alpestris* (Table 1), and identical to the other Palaearctic species of the genus.

### *B. emmerichi* (Falkenström, 1936)

Published information: 2n = 52 + X0 (♂), XX (♀) (Angus et al. 2015). Mitotic chromosomes, arranged as karyotypes, are shown in Fig. 1f–k, n, o. The new preparations, from the Kangding area, (Fig. 1f–i) are much better than the Qinghai ones shown by Angus et al. (2015), with the chromosomes less condensed and the C-banding more clearly defined. They confirm the published features of the karyotype and also allow improved comparison of the karyotypes of *B. emmerichi* and *B. macedonicus* (Georgiev, 1959). Thus autosome pair 12 was stated to be more evenly metacentric in *B. emmerichi*, and comparison of the Sichuan male preparation (Fig. 1o, p) with a similarly elongate preparation of *B. macedonicus* from Karanikoličko ezero (Fig. 1m, n) and with Cretan *B. macedonicus* (Fig. 1q, r) confirms this. Similarly, the smaller size of autosome pair 26 when compared with pairs 24 and 25 in *macedonicus* as against the more similar sizes of these three pairs in *emmerichi* is confirmed. However, the possibly smaller X chromosome in *emmerichi*, as was suggested by preparations of mitosis but not metaphase II of meiosis, is shown not to be the case. This demonstration that these are chromosomally distinct species is in complete agreement with the degree of difference shown by the COI DNA of the two species (Fig. 5).

**Figure 3.**
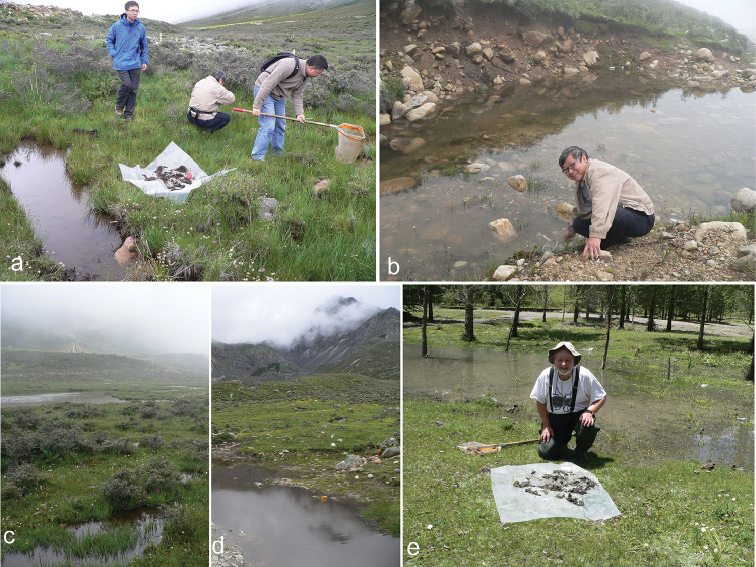
*B. emmerichi* collecting sites. **a**, **b** site 1, Yalashenshan, 4052 m a.s.l, **b** with Fenglong Jia **c** site 2, Yalashenshan, 4074 m **d** site 4, Yajiaheng, 3337 m a.s.l. **e** site 3, Boij ta, 3495 m a.s.l. with Robert Angus collecting.

**Figure 4.**
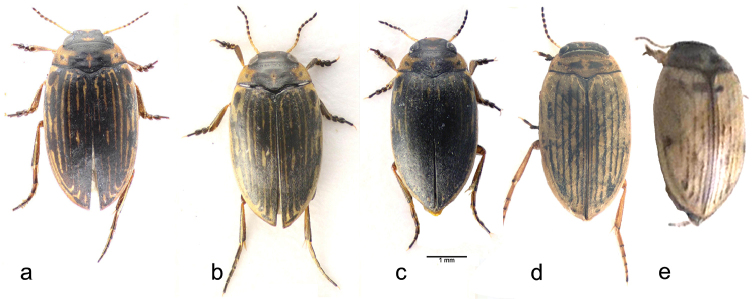
*B. emmerichi*, habitus. **a** lectotype **b**, **c** males from site 1, Yalashenshan **d** pale specimen from Maduo **e** the palest specimen seen, from the western Kun Lun mountains.

**Figure 5.**
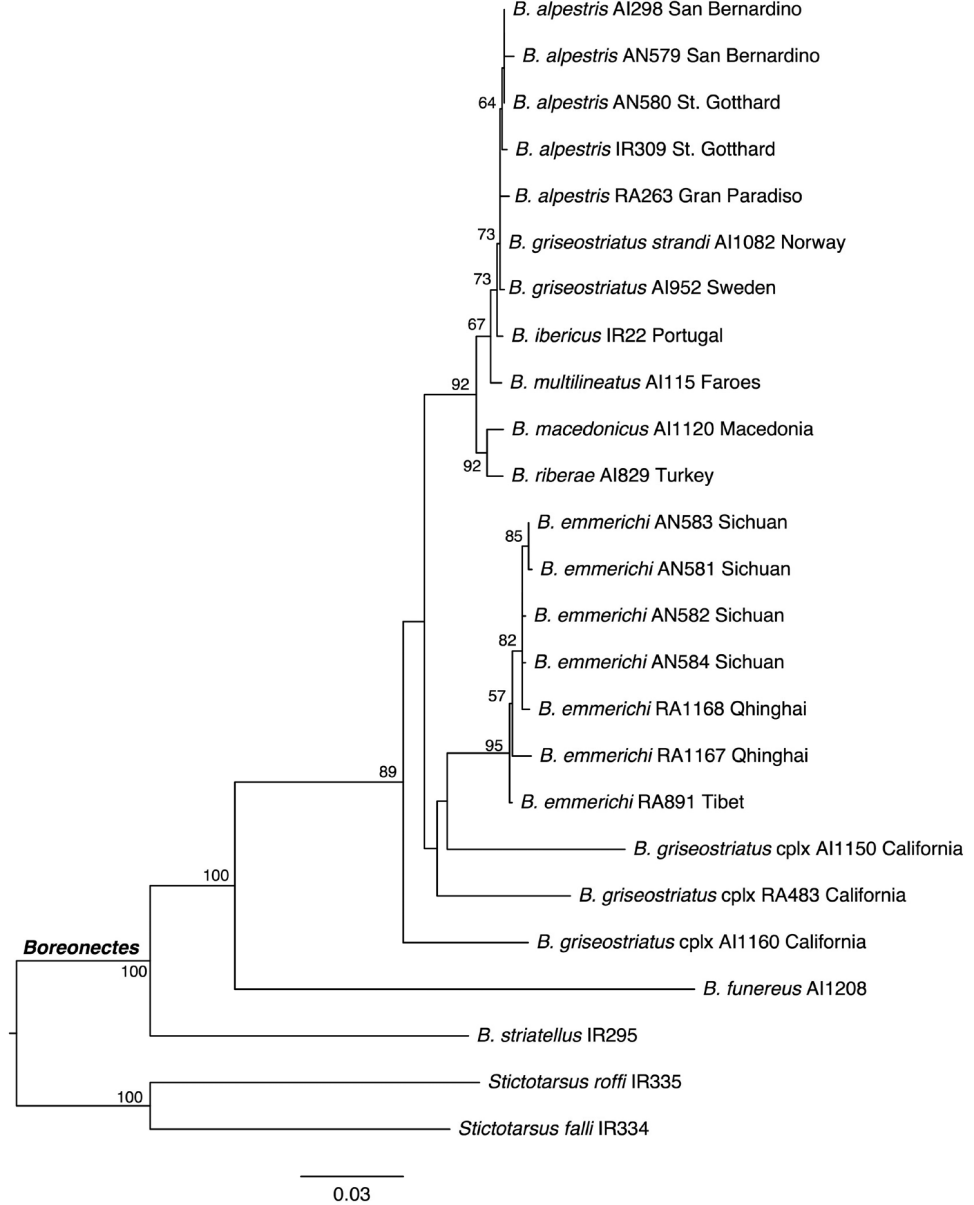
Phylogeny of the Palaearctic species of *Boreonectes*, obtained with maximum likelihood in RAxML using the combined mitochondrial and nuclear data. Numbers in nodes, bootstrap support. See Table 2 for details of the specimens.

### Variation within the species

#### *B. alpestris* (Dutton & Angus, 2007)

One of the surprises associated with collection of this species in 2016 was that the specimens from the St Gotthard pass appeared, even in the field, as noticeably larger than those from the San Bernardino. At the time it seemed that the St Gotthard specimens might be *B. griseostriatus* and those from the San Bernardino *B. alpestris*. In the event this proved not to be the case, but the size differences remain. Thus 10 ♂♂ from the San Bernardino range in length from 4.0–4.4 mm, with a mean length of 4.26 mm, while for 17 ♀♀from the same site the values are 5.2–5.1 mm, mean 4.38. From the St Gotthard the values are: 5 ♂♂, 4.3–4.6 mm, mean 4.51, and 8 ♀♀, 4.4–4.6 mm, mean 4.56 mm.

#### *B. emmerichi* (Falkenström, 1936)

As mentioned in the Introduction, the type material of *B. emmerichi* has more extensive darker markings, especially on the pronotum, than material from Qinghai, though it is matched by material from Sejilashan in southern Xizang (Angus et al. 2015). It was suggested that this more extensive darkening may be associated with wooded habitats. Latitude and longitude references for the type localities are, for Tatsienlu Tjiji 30°25' (=30.417°) N 102°40'(=102.667°) E (Balke 1992) and for Mukue Tatsienlu 30.05°N 102.03°E (Sykes et al. 2006). Google Earth gives altitudes of 1944 m a.s.l. for Tatsienlu Tjiji and 3935 m a.s.l. for Mukue Tatsienlu, with both localities in wooded areas. The localities from which *B. emmerichi* were collected in 2016 were: 1: Yalashenshan, 30.215°N 101.757°E, alt. 4052 m a.s.l.; 2: Yalashenshan, 30.205°N 101.755°E, alt. 4074 m a.s.l.; 3: Boij ta car parking area near Xinduqiao on the Kangding–Lhasa road, 30.048°N 101.569°E, alt. 3551 m a.s.l.; and 4: Yajiaheng, 29.927°N 101.995°E, alt. 3337 m a.s.l. Only the Boij ta locality (site 3) was in a wooded zone. In all localities water levels had risen recently due to the ongoing rain (see the silty water in Fig. 3e) but most of the pools had some aquatic vegetation. Only the pool shown in Fig. 3d had few beetles, suggesting that it was only recently flooded. All the chromosome preparations were from specimens collected at site 1. All this material has extensive dark markings. Fig. 4a shows the lectotype of *B. emmerichi*, while Fig. 4b, c shows two specimens from site 1. The specimen shown in "b" is a very close match for the lectotype and is the specimen from which the chromosomes shown in Fig. 1f, g were obtained. It seems clear that this more extensive darkening of the specimens is a regional phenomenon and not just a response to immediate local conditions.

Material from the northern part of the Tibetan Plateau is generally paler, and Fig. 4d shows a particularly pale specimen from Maduo, original shown as Fig. 1g by Angus et al. (2015). The palest specimen so far seen is from the western end of the Kun Lun mountains, taken by Ying Zhang. I have seen only a photograph of this specimen and this, though not good, does show the pattern (Fig. 4e).

#### A molecular phylogeny of the Palaearctic *B. griseostriatus* complex

The analysis of the combined mitochondrial and nuclear data recovered a monophyletic *Boreonectes* with strong support (Fig. 5), although due to the reduced number of outgroups its monophyly cannot be assessed adequately. The Palaearctic species, on the contrary, were not monophyletic, as *B. emmerichi* was found to be closer to some Nearctic specimens of the *B. griseostriatus* (De Geer, 1774) complex than to the western Palaearctic species, although with low support (Fig. 5). The Western Palaearctic species were recovered as monophyletic with strong support, and divided into the eastern Mediterranean *B. macedonicus* (Gerogiev, 1959) and *B. riberae* (Dutton & Angus, 2007) on one clade and all the western species on the other. Within the latter clade, species seem to be very close to each other, with poor resolution among them and no variation in the nuclear marker (H3, see above), suggesting a very recent expansion and differentiation between them. Contrary to the lack of genetic variation among the Western Palaearctic species of the group, the three studied Nearctic specimens show a high divergence, with ca. 13% of variable positions in the COI gene. There are no available data on the karyotypes of these Nearctic populations, but most probably they represent a complex of undescribed species.

**Figure 6.**
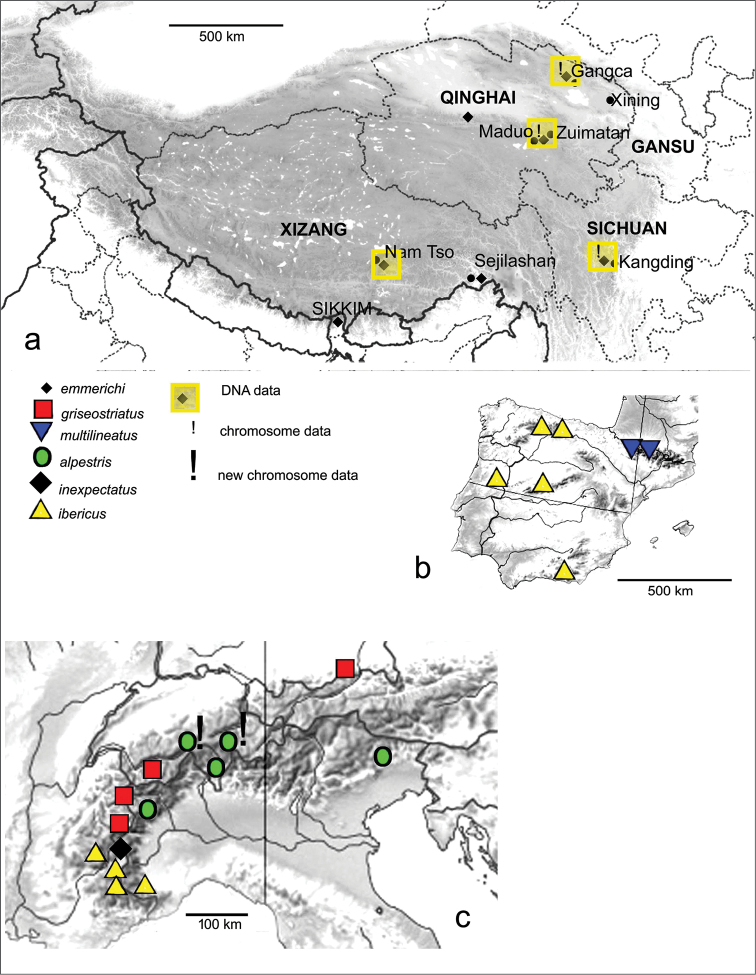
Maps of the distributions of various *B. griseostriatus* group species. **a** the Tibetan Plateau showing the localities of studied material of *B. emmerichi* **b** the Iberian Peninsula with the distributions of *B. ibericus* and *B. multilineatus* **c** The Alps with the distributions of *B. ibericus*, *inexpectatus*, *griseostriatus* and *alpestris*.

The Palaearctic species show a clearly uneven distribution of their diversity (Fig. 6). Thus, all the material from the Tibetan Plateau is *B. emmerichi*, with only limited variation in COI haplotypes. This can be compared with the situation in the Iberian Peninsula, occupying an area similar in size to the eastern part of the Tibetan Plateau, from which the *B. emmerichi* data have been obtained. Here again only one species, *B. ibericus* (Dutton and Angus 2007) is present, and this species has a wider distribution, also occurring in the French Alpes-Maritimes and Italian Alpi Marittime (Dutton and Angus 2007; Angus 2008), on Corsica (Angus 2010a) and in the Middle Atlas of Morocco (Angus 2010b). The occurrence of *B. multilineatus* (Falkenström, 1922) in the Pyrenees (Angus 2010b) reflects higher diversity, and this species is also known from inland Sweden and the British Isles (Dutton and Angus 2007) and, from COI data, from the Faroe Islands. The COI of this species appears sufficiently distinct (Fig. 5) to give confidence to the identification, although the study of more material is needed. However, the centre of high diversity is the Alps, where four chromosomally distinct species occur. One of these, *B. inexpectatus* is known only from a single lake, the Lac de Lauzet Inférieur (Dutton and Angus 2007) but the other three, *B. ibericus*, *B. griseostriatus* and *B. alpestris*, are more widely distributed, though apparently always allopatric. It is possible that the diversity of this group of species in Western Europe is, in part at least, a result of faunal movements associated with Pleistocene climatic fluctuations. *B. griseostriatus* group species are known as fossils in deposits dating from the Last Glaciation in the English Midlands (e.g. Coope et al. 1961), as well as from Starunia in the Ukrainian Carpathians (Angus 2010a), both areas outside the current known ranges of these species.
